# Desiccation tolerance in peatland desmids: a comparative study of *Micrasterias thomasiana* and *Staurastrum hirsutum* (Zygnematophyceae)

**DOI:** 10.1007/s00709-025-02061-1

**Published:** 2025-04-03

**Authors:** Y. Nemcova, J. Neustupa, M. Pichrtová

**Affiliations:** Department of Botany, Charles University, Benatska 2, 128 00 Prague 2, Czech Republic

**Keywords:** Chlorophyll fluorescence, Desiccation tolerance, Desmids, Peat bog, Ultrastructure, Zygnematophyceae

## Abstract

Desmids are valuable bioindicators in peatland ecosystems due to their sensitivity to environmental changes. In temperate and boreal wetlands, seasonal desiccation of aquatic habitats, which is increasing in frequency and severity due to ongoing climate change, is currently considered a key factor structuring the distribution of individual taxa. In this study, the desiccation tolerance of *Micrasterias thomasiana* and *Staurastrum hirsutum* isolated from contrasting hydrological environments in the peatland habitats of the Ore Mountains, Czech Republic, is investigated. Using controlled experimental conditions, we subjected both young, actively growing and old, mature cultures to four different desiccation treatments and evaluated morphology and photosynthetic performance. Our results showed that young and old cultures of both species exhibited a very similar photophysiological response. Severe desiccation led to an irreversible decline in the effective quantum yield of photosystem II in both species, resulting in cell death. Mild drought stress allowed the cultures to recover, indicating that the stress severity determines the recovery potential. Finally, prolonged desiccation resulted in irreversible damage in older cultures of both species, emphasizing the limited desiccation resilience of desmids. We observed similarities in morphology with *Zygnema* “pre-akinetes,” but in contrast to these resilient cells, the old cells of *M. thomasiana* and *S. hirsutum* did not survive the harsher desiccation conditions. Long-term mild desiccation revealed a higher resistance of *S. hirsutum*, probably due to the protective role of its dense mucilage. In nature, these two species usually inhabit localities with low desiccation risk or avoid and mitigate desiccation stress through localized survival strategies.

**Supplementary Information:**

The online version contains supplementary material available at 10.1007/s00709-025-02061-1.

## Introduction

Desmids (Desmidiales, Zygnematophyceae) are one of the most common groups of protists in the phytobenthos of peatlands. They are very sensitive to changes in the environment, and individual species have clearly defined ecological niches. Therefore, desmids are generally accepted as valuable freshwater bioindicators (Coesel 2001).

The majority of the world’s peatlands is located within the boreal zone of the northern hemisphere. However, there are also smaller areas in temperate zones, especially in mountain ecosystems and regions with high annual precipitation (Tanneberger et al. 2017). In Central Europe, peatbogs, such as those in the Ore Mountains of the Czech Republic, are at the edge of their natural environmental constraints, especially with regard to the interplay between precipitation and evapotranspiration (Čižková et al. 2013). These acidic wetlands are currently exposed to remarkable environmental fluctuations due to ongoing climate change (Breeuwer et al. 2009), including prolonged high temperatures and shifts in precipitation patterns. During summer, water levels in the pools drop significantly, and many shallow pools dry up completely (Neustupa et al. 2023). As a result, organisms inhabiting the phytobenthos in these environments may be exposed to significant drought stress.

Water plays a crucial role in cellular metabolism and structural integrity of macromolecules, and dehydration imposes severe stress, leading to macromolecule aggregation, organelle disintegration, and death. Therefore, drought-tolerant organisms developed various stress resistance mechanisms to avoid or tolerate desiccation stress (reviewed, e.g., by Holzinger and Karsten 2013). Fully desiccation-tolerant plants were defined as being able to withstand drying until approximately 10% of the original water content remains, which corresponds to about 50% relative humidity (RH) at 20 °C or to the cellular water potential of 100 MPa (Alpert and Oliver 2002).

In Zygnematophyceae, the most effective protection mechanism is the formation of dormant zygospores. The middle cell wall layer (mesospore) of these dormant stages contains algaenan, a sporopollenin-like material that is proposed as an effective protection against desiccation (Permann et al. 2022). Nevertheless, various members of Zygnematophyceae were found to be stress resistant in their vegetative state as well (Aigner et al. 2013; Pichrtová et al. 2014; Herburger et al. 2015). Aggregation of cells, mucilage production, accumulation of osmotically active compounds, and chloroplast photoprotection are the most common strategies of desiccation resistance in Zygnematophyceae (Holzinger and Karsten 2013; Holzinger and Pichrtová 2016). The biochemical composition of the cell wall has been found to be important for adaptation to cell dehydration, e.g. a high content of pectic component homogalacturonan increases cell resistance to dehydration-induced stress in *Zygnema* sp. (Herburger et al. 2019). A modification of the polar lipid profile in the thylakoid membranes also represents a biochemical adaptation to desiccation in *Zygnema circumcarinatum* (Arzac et al. 2023).

Numerous studies on the genus *Zygnema* have shown their ability to survive various stress conditions as pre-akinetes (Pichrtová et al. 2014, 2016; Trumhová et al. 2019). Pre-akinetes can be described as senescent or “stationary phase” vegetative cells filled with storage materials and with reduced photosynthetic activity in contrast to young and actively growing vegetative cells (Herburger et al. 2015). They also have thick cell walls with increased pectic content (Herburger et al. 2019). The pre-akinete formation was observed in natural conditions at the end of the growing season, and in cultures, it can be triggered by nitrogen depletion (Pichrtová et al. 2014, 2016). However, pre-akinetes can survive rapid desiccation in air at 10% relative humidity only after being acclimated through slow desiccation achieved either by controlled desiccation at high relative humidity or by pre-cultivation on solid agar plates (Pichrtová et al. 2014). Similarly, pre-acclimation in *Zygnema circumcarinatum* was reflected in the intensity of its transcriptional response (Rippin et al. 2017).

The formation of stress-tolerant senescent vegetative cells is a widespread phenomenon known from other algae, e.g. *Klebsormidium* (Morison and Sheath 1985) or *Tribonema* (Nagao et al. 1999). In the desmid genus *Micrasterias*, physiological resting stages referred to as ‘akinetes’ or even ‘winterforms’ were described (Meindl et al. 1989). The desiccation tolerance of desmids has not yet been investigated. However, Lütz-Meindl (2016) has summarized the current state of knowledge on the response of *Micrasterias* to various abiotic stress scenarios (e.g. high temperature, UV radiation, high salinity, and heavy metal exposure). These unfavorable environmental conditions (including desiccation) cause oxidative stress associated with the production of reactive oxygen species. Experimental application of H_2_O_2_ resulted in severe physiological and ultrastructural alternations in *M. denticulata* (Darehshouri et al. 2008).

Although experimental studies on the desiccation tolerance of individual desmid taxa are lacking, we can gain some information from studying the distribution of desmids in natural habitats. Neustupa et al. (2024) studied the composition of desmids in ombrogenous bog pools on a gradient ranging from pools that were dried out throughout the summer season to those that were constantly flooded. They showed that a prolonged desiccation period significantly affects species composition. However, most species tended to occur at sites that did not dry out completely during the summer. *Cosmarium obliquum* was the only species that preferred pools with a longer desiccation period (Neustupa et al. 2024). Coesel (1982) suggested that adaptation to periodic desiccation could explain the variations in body shape among desmid communities. This includes a range from flattened and elongated forms with higher surface-to-volume (S:V) ratio to cylindrical forms with lower S:V ratio, correlating with the increasing atmospheric influence on their habitats. Similarly, Neustupa et al. (2011) found that desmids with low S:V ratios tended to survive better in the drier environments of European peat bogs.

In this study, we investigated the desiccation resistance of two common desmid species *Micrasterias thomasiana* and *Staurastrum hirsutum*, in experimental conditions*.* These species were isolated from two nearby Ore Mountains habitats (Czech Republic). While *M. thomasiana* was isolated from a minerotrophic pool fed by groundwater with a relatively stable water level, *S. hirsutum* was isolated from a shallow, ombrogenous pool, fed by precipitation with fluctuating water regime. Both species were abundant in phytobenthos of Ore Mountains peatland pools. We asked whether different hydrological regimes of their original localities result in differences in their stress resistance. We investigated both species’ morphological and photophysiological responses to desiccation stress and recovery. Moreover, we hypothesized that old, mature cells in the stationary phase are more resistant to desiccation compared to actively growing vegetative cells, as was observed in *Zygnema* (Pichrtová et al. 2014).

## Material and methods

### Collection, isolation, and cultivation of strains

A clonal strain of *Micrasterias thomasiana* was obtained as a single-cell isolate from the phytobenthos of a minerotrophic pool in a peatland headwater area of a levelled mountainous plateau in the Ore Mountains (near Skelný vrch Peak), Czech Republic, 50.509861N, 13.196075E, pH 5.3 (Neustupa and Woodard 2024) in April 2022. Similarly, *Staurastrum hirsutum* was isolated in the same area from a different pool (50.5018619N, 13.2076936E; pH 4.2) in June 2022.

Before the experiments, the biomass was grown in 50-ml Erlenmeyer flasks in liquid DYV medium (Andersen 2005) and kept in 24 °C and illumination of 50 μmol⋅m^−2^⋅s^−1^ (TLD 18W/33 fluorescent lamps, Philips, Amsterdam, the Netherlands). Light intensity was measured with a Walz ULM-500 radiometer equipped with Spherical Micro Quantum Sensor. For the experiments we used young, fresh cultures (3 weeks after re-inoculation into the fresh medium) and old, stationary phase cultures that were kept for a longer period (5 months) without re-inoculation or adding fresh medium. We used 5-month-old cultures because *Zygnema* strains have been shown to develop resistant pre-akinetes full of reserve substances with increasing age of the cultures, which were able to resist various stress factors (Herburger et al. 2019, Pichrtová et al. 2014, 2016; Trumhová et al. 2019).

### Light and fluorescent microscopy, Nile red staining, auramine vital staining

Algal cells from young and old cultures were observed with an Olympus BX51 light microscope equipped with Nomarski differential contrast. Mucilage was observed after addition of diluted Indian Ink (Winsor & Newton, London). Images were captured with Olympus Camedia C-5060Z digital microphotographic equipment (Olympus, Tokyo, Japan). Nile red (9-diethylamino-5H-benzo[a]phenoxazine-5-one) with excitation/emission maxima of 552/636 nm is the most commonly used fluorescent lipophilic stain for intracellular triacylglycerol (TAG) detection in microalgae. Forty microliters of stock solution (250 µg Nile red (Carl Roth GmbH, Karlsruhe, Germany) in 1 ml of acetone) was added to 4 ml of culture media containing algae (final NR concentration 2.5 µg/ml). Staining was performed for 10 min. Stained cells were visualized with fluorescent microscope (Olympus BX51) using the WGS mirror cube (excitation wavelength 510–550 nm, emission wavelength 590 nm). Microphotographs were taken with an Olympus DP72 camera (Olympus, Tokyo, Japan). The approx. quantification of the lipid bodies was coded using fluorescent microscopic images. Colors were inverted, and images were converted to black and white in Adobe Photoshop CS3 (2007 Adobe Systems Incorporated). The ratio between cytosolic lipid body content and cell content was counted for five cells in Image J 1.53e. Auramine staining was performed in a pilot experiment comparing old, mature cells with young, actively growing vegetative cells (data not shown), as described in Trumhová et al. (2019). In brief, cells were stained in an aqueous solution of auramine O at concentration of 0.1% (Sigma-Aldrich, Steinheim, Germany) for 10 min in the dark. Stained cells were observed with fluorescent microscope (Olympus BX51) using the WBS mirror cube (excitation wavelength 450–480 nm, emission wavelength 520 nm). Auramine O stains the endomembrane system of metabolically active cells in a bright yellow-greenish color (Hawes and Davey 1989). In our case, cells with contracted chloroplasts were stained brighter than actively growing cells, so we did not use this method and performed a light microscopic assessment of viability after 12 days, when dead cells were clearly recognizable.

### Flat/pellet embedding on agarose, transmission electron microscopy

Flat embedding of *Micrasterias thomasiana* was carried out according to the procedure described in Wenzel et al. (2021). Briefly, 15 µl of cell suspension was spotted onto a 0.25-mm-thick square film of 1.5% agarose (thickness was controlled using Gene Frame AB0576, Thermo Fischer Scientific). Excess liquid was removed with a Whatman filter paper (Grade 1). This step was repeated until approximately 10 cells were placed on the agarose film. Wash-off from the surface was effectively prevented by enclosing the cells in an agarose sandwich. The sample was covered with a second thin layer of 1.5% agarose (cooled to ~ 50 °C before application). The fresh agarose spot (25 µL) was immediately covered with a glass coverslip and pressed with a small weight (25 g) to create a thin and flat surface. After ~ 1 min, the weight was removed, and the coverslip gently slid off the agarose, resulting in a flat and stable agarose sandwich. Pellet embedding was used for *Staurastrum hirsutum*. The cells were pelleted by centrifugation (5500 rpm, centrifuge FC5760 with rotor angle, Ohaus Europe GmbH, Switzerland), mixed with agarose (~ 50 °C) and cut into 2 × 2 mm blocks after cooling.

For transmission electron microscope observations, the mounted cells were fixed for 2 h temperature in a 2% glutaraldehyde solution in 0.1 mol/L cacodylate buffer containing 2.7% glucose at room temperature overnight at 5 °C and then post-fixed for 12 h at 5 °C in 1% osmium tetroxide in the same buffer. After dehydration through an ethanol series (70%, 96%, 100%), the cells were embedded in Spurr’s medium (Spurr 1969) over butanol. Ultrathin sections cut with a diamond knife on an Ultracut E (Reichert-Jung, Vienna, Austria) were poststained with lead citrate and examined with a JEOL 1011 TEM (JEOL Ltd., Tokyo, Japan). Photomicrographs were taken with a Veleta CCD camera with image analysis software (Olympus Soft Imaging Solution GmbH).

### Design of the desiccation experiments

A series of four desiccation experiments were conducted to test resistance and potential acclimation in cultures of *S. hirsutum* and *M. thomasiana*. The desiccation experiments were performed in custom-designed plexiglass chambers following the same principles as those of Pichrtová et al. (2014). Briefly, the chamber contained 250 ml of saturated NaNO_3_ solution, and above this solution, separated by a plastic grid, biomass was placed on glass fiber filters (Whatman GF/C, 47 mm). The chamber was closed and sealed to allow the relative air humidity above the NaNO_3_ solution to stabilize at the theoretical value of 74% RH at 25 °C (Greenspan 1977). After rehydration of the desiccated samples, the wet filters were placed in another chamber over 250 ml of distilled water.

In experiment I, young and old cultures were centrifuged, and 4 × 30 µL of the concentrated material was pipetted onto glass fiber filters and transferred to the desiccation chamber. The samples were immediately re-hydrated with DYV medium after the effective quantum yield (Φ_PSII_) had reached values below 0.1, i.e., 5 h after the start of the experiment. Measurement of the effective quantum yield and observation of the cell morphology by light microscopy were also performed during the recovery phase of 7 days.

Experiment II was designed to provide milder stress than experiment I. 4 × 30 µl of the centrifuged biomass (young and old cultures) was also pipetted onto the filters, and the filters were additionally moistened by adding 500 µl of distilled water. This experiment aimed to test how the cells from liquid cultures can survive on the filters when exposed to the air at 74% RH for several hours. The samples were re-hydrated with DYV medium 13 h after the start of the experiment. Φ_PSII_ was then measured 19, 40, and 58 h after rehydration (32, 53, and 71 h after the start of the experiment, respectively).

Experiment III was conducted in parallel with experiment II. An additional set of samples (4 × 30 µl) of old cultures was placed in a different desiccation chamber and treated in the same way. However, rehydration was postponed for 22 h after the start of experiment when the Φ_PSII_ values decreased considerably. The aim of the experiment was to test the potential effects of acclimation in old cells, as we assumed that these cells in stationary phase are more resistant to desiccation.

Experiment IV: Samples of young cultures on the filters from experiment II were used for further investigation. The wet filters with the biomass samples were kept in chambers in very humid air (above water, 98–100% RH) and repeatedly moistened for 14 days. They were then kept under the same conditions, but without further remoistening, for a further 13 days and then re-hydrated again. After the last Φ_PSII_ measurement, two drops of each species of *S. hirsutum* and *M. thomasiana* were cut from the filter and transferred to an Erlenmeyer flask containing 30 ml of fresh DYV medium. The viability of the culture was checked after 10 and 30 days.

### Chlorophyll a fluorescence measurement

Chlorophyll fluorescence was measured throughout the experiments to assess the photosynthetic activity of the experimental cultures. We measured the relative parameter of effective quantum yield (Φ_PSII_), which is related to the photochemical energy conversion effectivity in photosystem II in a light-adapted state (Roháček and Barták 1999). This parameter is considered a good eco-physiological indicator of how plants respond to environmental stress (Rascher et al. 2000). Φ_PSII_ is computed as (F_M_′-F)/F_M_′, where *F* is the steady state fluorescence and *F*_M_′ is the maximum fluorescence in the light-adapted state, measured after application of a saturation pulse. The desiccation chamber with the biomass on filters was continuously illuminated at 45 μmol m^−2^ s^−1^, and measurements were performed non-invasively through the transparent lid. We used an imaging modulated fluorimeter FluorCam (Photon System Instruments, Czech Republic).

Four independent sample replicates per strain were used to measure the effective quantum yield. The two-way analysis of variance (ANOVA) was used to evaluate the effects of strain age and species identity on their initial levels of effective quantum yields in experiments I and II. The analysis was conducted in PAST, ver. 4.10 (Hammer et al. 2001).

## Results

### Cultures entering the experiment

**Fig. 1:**
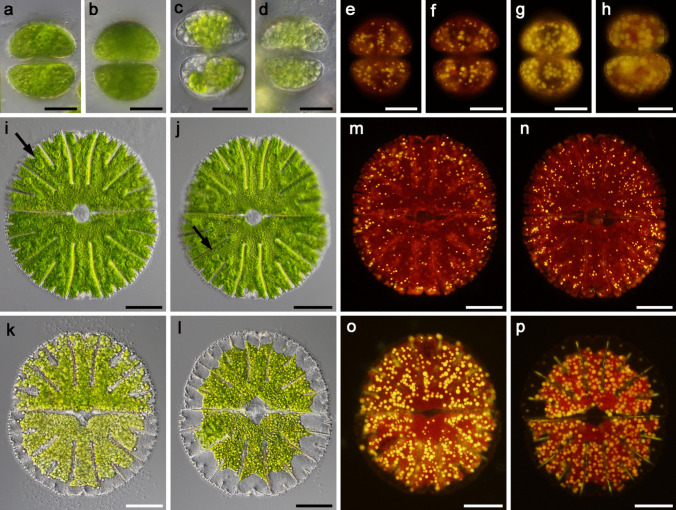
Morphology of *Staurastrum hirsutum* and *Micrasterias thomasiana* entering the experiments. **a**–**h** *S. hirsutum* **a**,** b** cells from actively growing, 3-week-old culture, viewed under a light microscope (LM); **c, d** cells from mature, 5-month-old culture, LM; **e, f** Nile red stained cells from young culture, viewed under a fluorescence microscope (FM); **g, h** Nile red stained cells from old culture (FM), lipid bodies containing triacylglycerol are visualized). **i–p** *M. thomasiana* **i, j** cells from 3-week-old culture (LM), pyrenoids are marked with arrows, the nucleus is located between the two semicells; **k, l** cells from mature 5-month-old cultures (LM), in some cells a shrinkage of the chloroplast was visible; **m, n** Nile red stained cells from young culture (FM); **o, p** Nile red stained cells from old culture (FM). Bars 20 µm **a**–**h** and 50 µm **i**–**p**

**Fig. 2:**
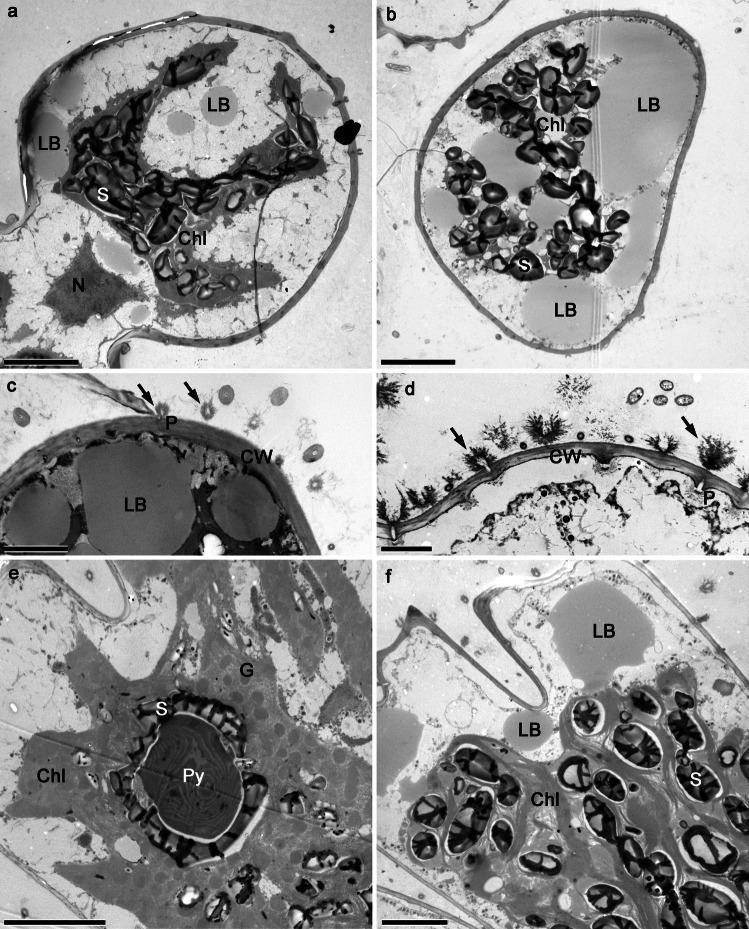
Transmission electron micrographs of *Staurastrum hirsutum* and *Micrasterias thomasiana* entering the experiments. **a–c** *S. hirsutum* (**a** longitudinal section through the cell from actively growing, 3-week-old culture bearing a three-lobed chloroplast and small lipid bodies, note the nucleus in the center of the isthmus; **b** transverse section through the cell from 5-month-old culture filled with large lipid bodies, the chloroplast contains numerous starch grains; **c** the cell producing mucilage from pores (arrows) in the cell wall). **d–f** *M. thomasiana* (**d** the cell producing mucilage from the numerous pores (arrows); **e** longitudinal section through the cell from young culture, chloroplast with thylakoids organized in grana, bearing pyrenoid, surrounded by starch grains; **f** section through the older cell with large lipid bodies in the cytoplasm and chloroplast containing numerous starch grains). Bars 5 µm (**a**, **b**, **e**, **f**) and 2 µm (**c**, **d**). Chl, chloroplast; CW, cell wall; G, grana; LB, lipid body; N, nucleus; P, pore; Py, pyrenoid; S, starch

For the experiments, we used young, fresh cultures (3 weeks after re-inoculation into the fresh medium) and 5-month-old, stationary phase cultures of the two desmid species *Staurastrum hirsutum* and *Micrasterias thomasiana. Staurastrum hirsutum* is a triradiate spinous desmid with cell dimensions ca. 45 × 35 µm. In young, actively growing cells (Fig. 1a–b), the chloroplast of each semi-cell forms three lobes, each of which reaches one of the cell corners (Fig. 2a). The chloroplast contains several pyrenoids surrounded by starch grains, and numerous starch grains are also scattered within the chloroplast. While the young cells contain only a few small cytosolic lipid bodies, the lipid body content estimated from fluorescent images of five cells was 4.4%; standard deviation 0.007 (Fig. 1e–f, Fig. 2a), the cells of the old cultures (Fig. 1c–d) are full of lipid bodies containing triacylglycerols, with an estimated lipid body content of 28.9%, standard deviation 0.07 (Fig. 1g–h, Fig. 2b–c). In the old cells, the chloroplast is often compressed by large lipid bodies. The dividing cells remain in a common firm mucilage, that is released through the pores in the cell wall (Fig. 2c). The cells embedded in mucilage form macroscopic densely packed clumps (Suppl. Figure 1a, stained with Indian Ink).

*Micrasterias thomasiana* (cell dimensions ca. 240 × 220 µm) is typical for biradial symmetry, i.e., each cell consists of two flattened semi-cells. Each semi-cell is further divided into a polar lobe and two lateral lobes that are further subdivided into the fourth order. In actively growing cells, the chloroplast fills almost the entire cell volume (Figs. 1i–j, Fig. 2e). Conspicuous pyrenoids are surrounded by starch, which sometimes fuses into a sheath; numerous starch grains are scattered within the chloroplast. Lipid bodies are small and more densely distributed around the cell perimeter, and the lipid body content estimated from fluorescent images of five cells was 2%, standard deviation 0.004 (Fig. 1m–n). Older cells sometimes have retracted chloroplasts (Fig. 1k–l) with densely packed starch grains (Fig. 2f) and larger lipid bodies with an estimated lipid body content of 15.6%, standard deviation 0.02 (Fig. 1o–p, Fig. 2f). The dividing cells remain in a common thin mucilage, in which the cells are connected but not densely packed (Suppl. Figure 1b–c, stained with Indian Ink). Enormous amounts of mucilage are actively released from the pores of the cell wall (Fig. 2d).

### Desiccation experiments

**Fig. 3:**
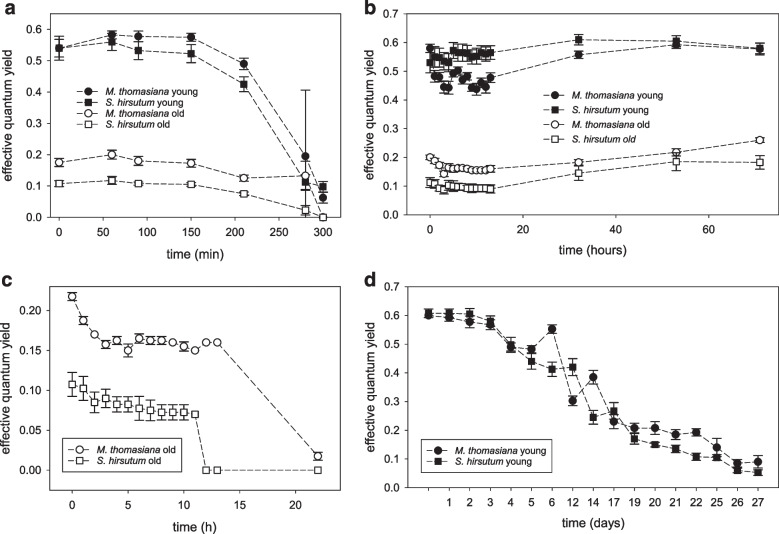
Effective quantum yield (Φ_PSII_) in a response to different desiccation scenarios. Severe desiccation stress, 74% RH (experiment I) **a** Mild drought stress 74% RH + additional moistening of the filter (experiment II). **b** Prolonged mild desiccation of old cultures (experiment III). **c** Pro-longed period on wet filters of young cultures, followed by mild desiccation stress (above water, 98–100% RH). **d** Results are means ± standard deviations of four independent replicates

**Fig. 4:**
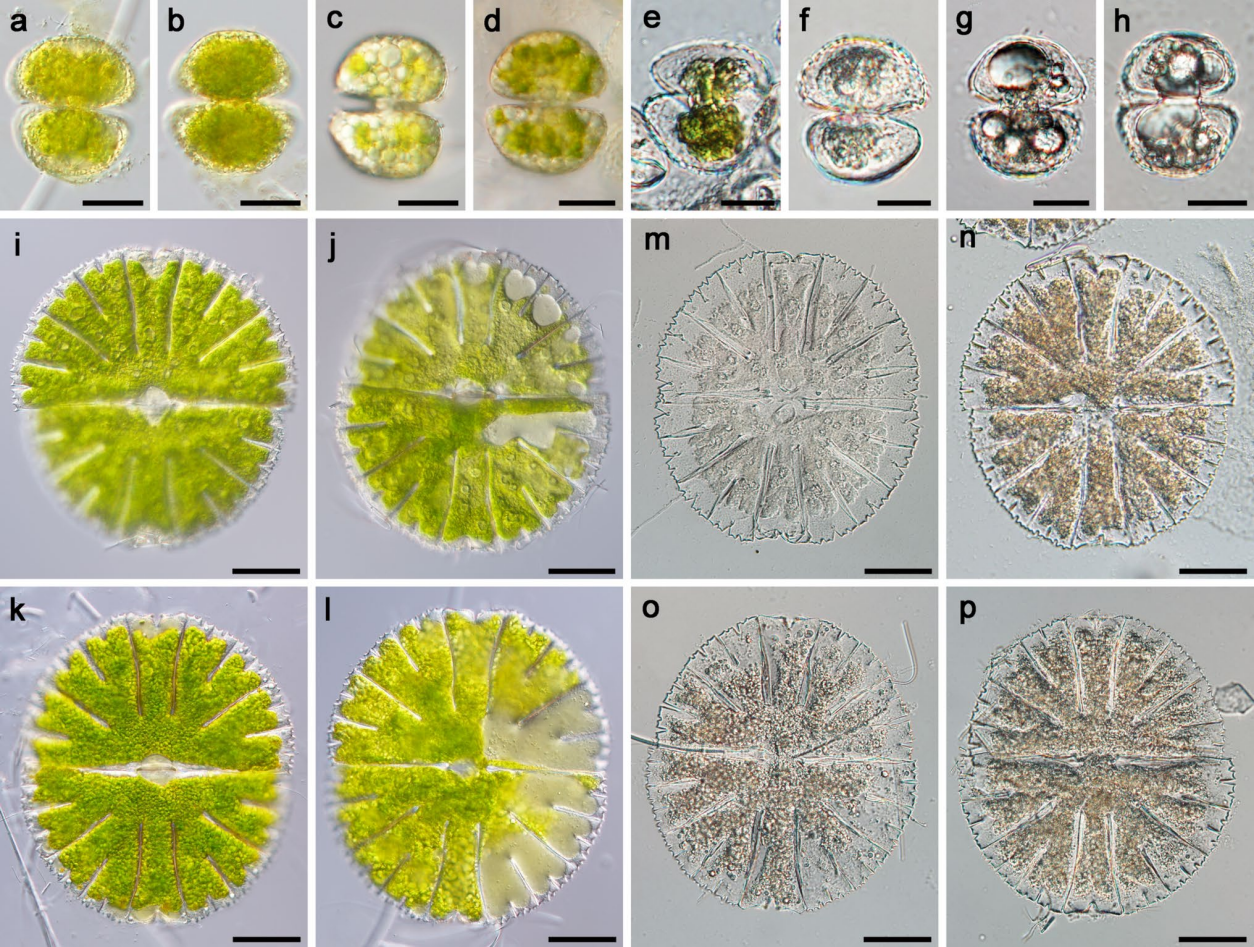
Morphology of *Staurastrum hirsutum* and *Micrasterias thomasiana* after fast severe desiccation (experiment I). **a**–**h** *S. hirsutum* (**a, b** cells from 3-week-old culture (young cells) 2 days after rehydration; **c, d** cells from 5-month-old culture (old cells) 2 days after rehydration; **e, f** young cells 12 days after rehydration, note gradual chloroplast bleaching; **g, h** old cells 12 days after rehydration full of large lipid droplets). **i–p** *M. thomasiana* (**i, j** young cells 2 days after rehydration, note beginning of irreversible damage to the cell resulting later in cell death; **k, l** old cells 2 days after rehydration; **m, n** young cells 12 days after rehydration; **o, p** old cells 12 days after rehydration). Bars 20 µm (**a**–**h**) and 50 µm (**i**–**p**)

Experiment I—severe desiccation: Young cultures of both species had a very similar photosynthetic performance (Fig. 3a), with an initial mean effective quantum yield of 0.54. In contrast, old cultures of both species showed minimal Φ_PSII_ values (between 0.1 and 0.2) even before the desiccation experiment started. After 210 min, all measured values dropped considerably. The samples were re-hydrated after 300 min, when Φ_PSII_ dropped below 0.1 in the young cultures and to zero in the old cultures. Markedly, none of the tested cultures survived this treatment, and Φ_PSII_ values were not recovered even after 7 days of observation (data not shown). Two days after the experiment, both the young and old cells of *S. hirsutum* were greenish yellow (Fig. 4a–b and c–d, respectively). Later, however, there was a gradual contraction of the chloroplast (Fig. 4e), followed by chlorophyll degradation. Dead young cells (Fig. 4f) and old cells full of fused lipid bodies (Fig. 4g–h) containing triacylglycerol were found on all filters 12 days after the experiment. The performance of *M. thomasiana* cells was very similar (Fig. 4i–p). After rehydration, there was a gradual chlorophyl degradation within the chloroplast, which resulted in only half of the chloroplast being green (not shown) and subsequently the entire chloroplast in dead cells turned brown. The ANOVA model confirmed that differentiation between the young and old strains were key to their initial photosynthetic performance (Table 1). In both taxa, the young populations exhibited significantly higher Φ_PSII_ values. In addition, the two studied taxa slightly differed in their initial Φ_PSII_ values yielding a weakly significant *p*-values for the effect of “species” and the interaction of two fixed factors.

**Table 1:** Results of two-way ANOVA evaluating variation of initial quantum yields in clonal populations of *Staurastrum hirsutum* and *Micrasterias thomasiana* exposed to severe desiccation (experiment I)

Source	df	SS	MS	*F*	*p*
Age	1	0.6320	0.6320	1140.00	0.0001
Species	1	0.0036	0.0036	6.49	0.0255
Age:species	1	0.0049	0.0049	8.84	0.0116
Residual	12	0.0067	0.0006		
Total	15	0.6472			

*df* degrees of freedom, *SS* sum of squares, *MS* mean squares, *p* probability of the null hypothesis

**Fig. 5:**
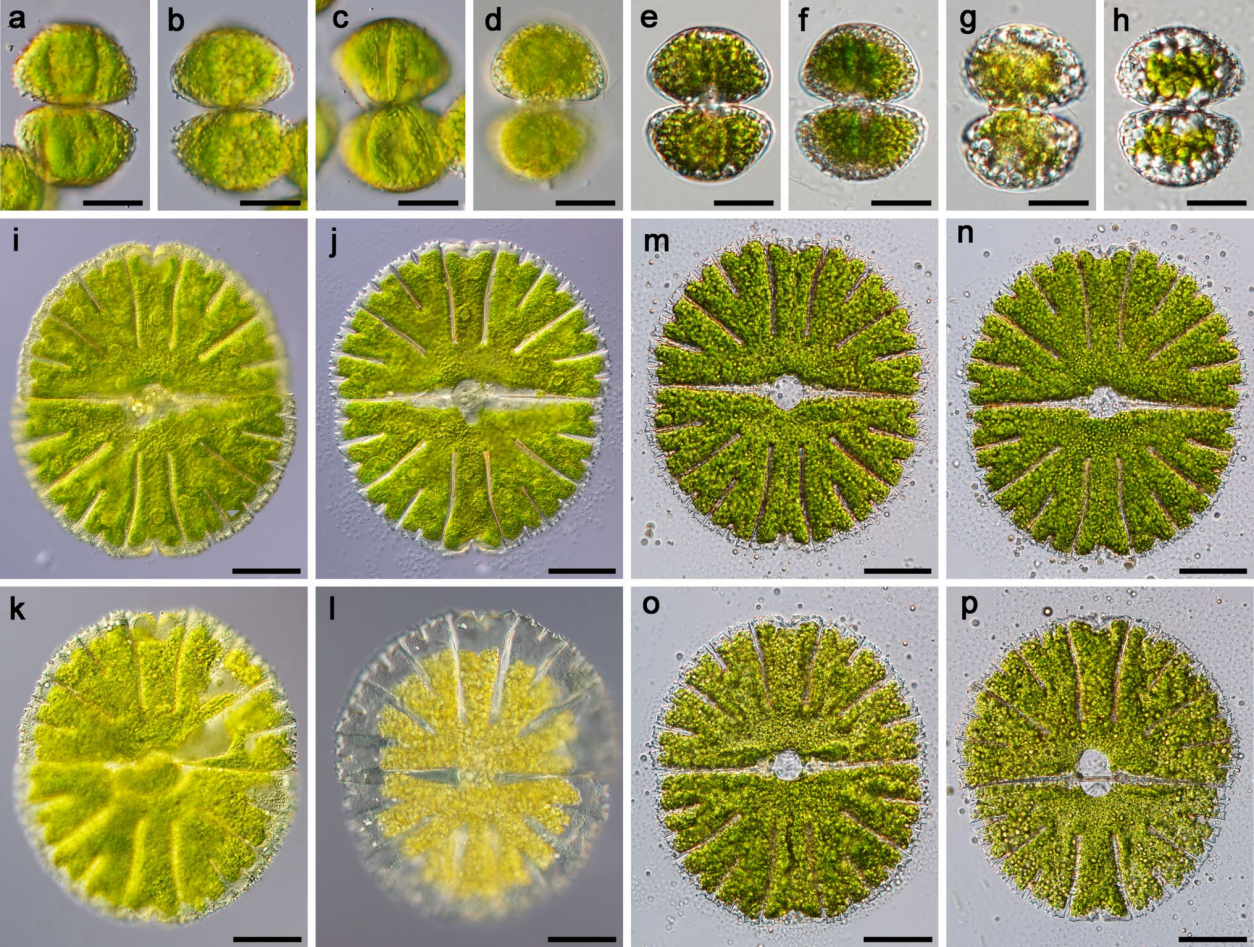
Morphology of *Staurastrum hirsutum* and *Micrasterias thomasiana* after mild drought stress (experiment II). **a**–**h** *S. hirsutum* **a, b** cells from 3-week-old culture (young cells) 2 days after rehydration; **c, d** cells from 5-month-old culture (old cells) 2 days after rehydration; **e, f** young cells 12 days after rehydration; **g, h** old cells 12 days after rehydration (full of storage material). **i–p** *M. thomasiana* **i, j** young cells 2 days after rehydration; **k, l** old cells 2 days after rehydration; **m, n** young cells 12 days after rehydration; **o, p** old cells 12 days after rehydration). Bars 20 µm **a**–**h** and 50 µm **i**–**p**

**Fig. 6:**
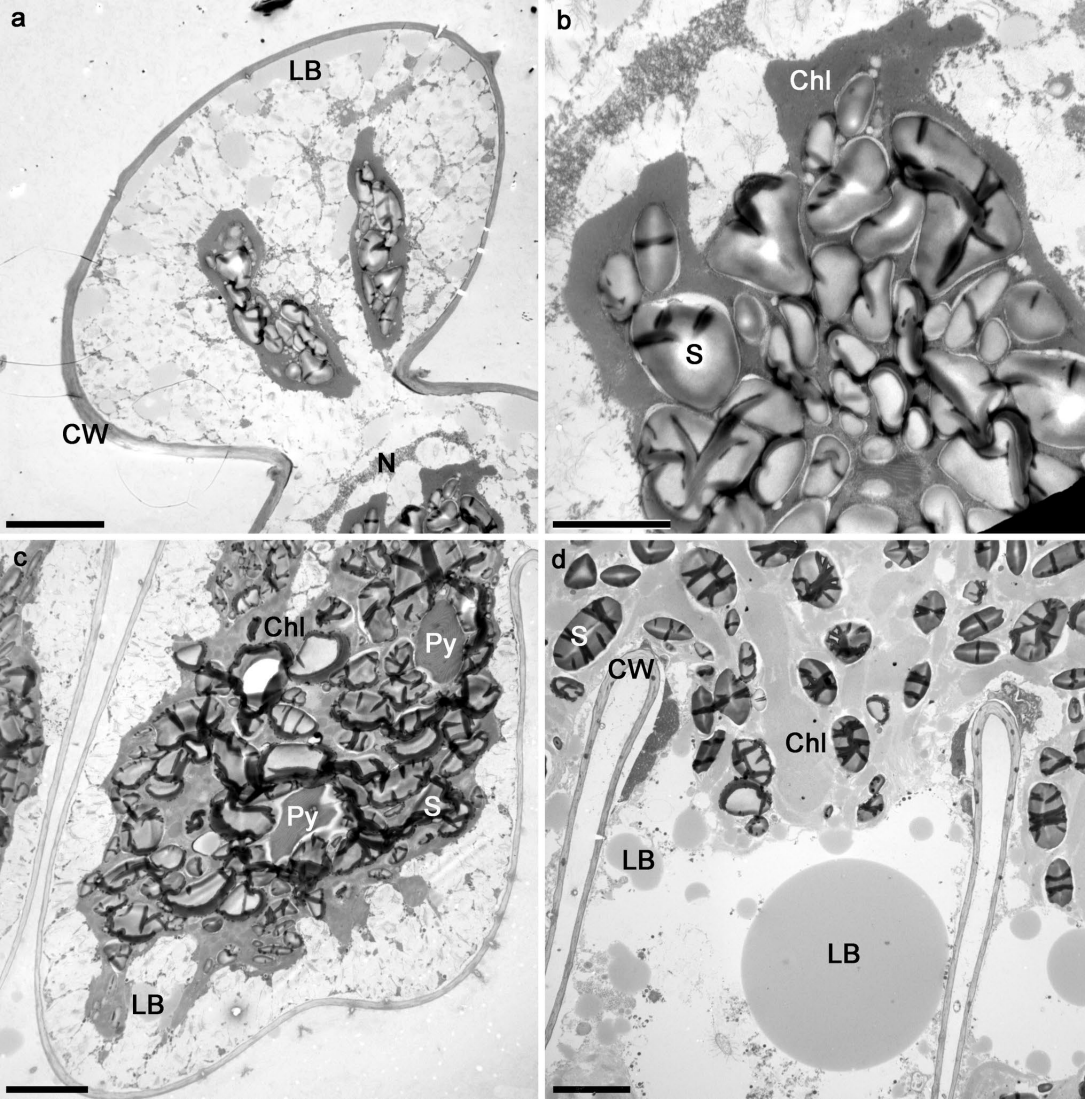
Transmission electron micrographs of *Staurastrum hirsutum* and *Micrasterias thomasiana* after mild drought stress desiccation (experiment II) 2 days after rehydration. **a–b** *S. hirsutum* **a, b** the cell from 3-week-old culture (young cell), the chloroplast contains densely packed starch granules). **c**–**d** *M. thomasiana* (**c** the young cell, note partially shrink chloroplast and pyrenoids with less organized starch sheath, **d** the old cell with large and small lipid droplets). Bars 5 µm **a**, **c**, **d** and 2 µm **b**. Chl, chloroplast; CW, cell wall; LB, lipid body; N, nucleus; Py, pyrenoid; S, starch

Experiment II—mild drought stress: The filters with the biomass additionally moistened with distilled water were kept at 75% RH for 13 h, and although the filters appeared dry at the end, the effective quantum yield decreased only slightly (Fig. 3b). As in experiment I, the initial Φ_PSII_ values of young and old cultures differed considerably. After rehydration, Φ_PSII_ increased in all cultures (Fig. 3b). Microscopic observation also confirmed that all cultures (young and old) of the two species tested, *S. hirsutum* (Fig. 5a–h; Suppl. Figure 2a, c) and *M. thomasiana* (Fig. 5i–p; Suppl. Figure 2b, d), were able to survive this treatment. In general, the young cells of *S. hirsutum* appeared more granular after desiccation treatment, and ultrastructural examination confirmed a higher content of densely packed starch granules (Fig. 6a–b). The cells of the old culture were lost during the TEM preparations. Similarly, the ultrastructure of both young and old cells of *M. thomasiana* did not change much after desiccation treatment. The only visible difference was that the starch granules, which formed a sheath around the conspicuous pyrenoids in the young cells, were no longer as precisely arranged after desiccation, their density increased, and in some cells a shrinkage of the chloroplast was visible (compare Figs. 2e and 6c). The old cells before and after desiccation were similar (compare Figs. 2f and 6d). The ANOVA model again showed that the age of the cultures was the single most important effect determining the initial Φ_PSII_ values in this experiment (Table 2). However, strongly significant *F*-value was detected for the effect of "species.” too. This result reflected consistently higher initial Φ_PSII_ values for *M. thomasiana* when compared to *S. hirsutum* populations.

**Table 2:** Results of two-way ANOVA evaluating variation of initial quantum yields in clonal populations of *Staurastrum hirsutum* and *Micrasterias thomasiana* exposed to mild drought stress (experiment II)

Source	df	SS	MS	*F*	*p*
Age	1	0.6360	0.6360	1394.00	0.0001
Species	1	0.0189	0.0189	41.44	0.0001
Age:species	1	0.0014	0.0014	3.08	0.1046
Residual	12	0.0055	0.0005		
Total	15	0.6618			

*df* degrees of freedom, SS sum of squares, MS mean squares, *p* probability of the null hypothesis

Experiment III—prolonged desiccation of old cultures: Old cultures kept in the desiccation chamber for 22 h and lost their photosynthetic activity at the end (Fig. 3c), and no cells were able to survive this treatment (Suppl. Figure 2e, f and Suppl. Figure 3a–e). Similar to experiment I, the cells were greenish yellow two days after rehydration. However, over the next 10 days, chlorophyll was degraded in both species, *S. hirsutum* and *M. thomasiana*. Many cells of *S. hirsutum* were full of fused lipid bodies (Suppl. Figure 3b).

Experiment IV—pro-longed period on wet filters, followed by mild desiccation stress: The young cultures from experiment II were kept on wet filters for pro-longed period of time. Morphology of cells 12 days after mild desiccation treatment is shown on Fig. 5e–f and m–n. Increased granularity of the cell content in both species in either young or old cultures was visible, caused probably by increased production of reserve substances and triacylglycerols. Although the filters were kept moist by regular rehydration, their Φ_PSII_ steadily decreased. After 14 days, when they were no longer hydrated, the decline of Φ_PSII_ continued. After the last rehydration on day 27, no measurable fluorescence signal could be detected (Fig. 3d). Nevertheless, the recultivation test showed that at least some cells of *S. hirsutum* survived this treatment, and the culture began to grow. A visible green coloration was present in the Erlenmeyer flask after 10 days, and a dense culture was present after 30 days of cultivation in fresh DYV medium. On the other hand, even after 30 days, no culture was established from cut filter paper with droplets of *M. thomasiana*.

## Discussion

In this study, we explored whether the two desmid species, *Micrasterias thomasiana* and *Staurastrum hirsutum*, which were isolated from two nearby habitats with different hydrological regimes, show differences in their responses to desiccation. As far as we know, this is the first study investigating desiccation tolerance in desmids in controlled experimental conditions. We followed a modified desiccation setup after Pichrtová et al. (2014), where the length of the desiccation phase in four *Zygnema/Zygnematopsis* species was determined by the operating efficiency of the photosystem II. Desiccation treatments continued until Φ_PSII_ dropped suddenly to zero or settled at an above-zero value. Similarly, in our experiment I, both young and old cultures of *M. thomasiana* and *S. hirsutum* were subjected to desiccation until photosynthesis ceased (Φ_PSII_ had reached values below 0.1) followed by rehydration with a fresh medium.

Our findings confirmed that desmids are highly sensitive to desiccation. As soon as their effective quantum yield fell below a critical value of 0.1, they were unable to recover. The cells were so severely damaged that they died within a few days. It is difficult to compare desiccation resistance of various species in different experimental set-ups that results in different desiccation rates. However, some members of Zygnematophyceae seem to be less adapted to periods of drought, e.g., *Mesotaenium endlicherianum *(Rieseberg et al. 2023) and *Zygnemopsis* sp. (Pichrtová et al. 2014) which did not recover their photosynthetic activity after Φ_PSII_ considerably dropped to values close to 0 (45 min at 64.5% RH and 8 min at 10% RH, respectively). On the other hand, several strains of *Zygnema* spp. and *Zygogonium ericetorum* are desiccation resistant (Aigner et al. 2013; Pichrtová et al. 2014, 2016; Herburger et al. 2015; Rippin et al. 2017). Previous studies have shown that desiccation resistance depends on the cell type. In *Zygogonium ericetorum*, two morphotypes have been identified: a purple morph, which contains secondary metabolites such as phenolics and tannins, and a green morph, which lacks dark coloration. It has been shown that the green morph is more resistant to desiccation compared to the purple morph (Aigner et al. 2013). *Zygnema* survives desiccation (and other stresses) in the so-called pre-akinetes, old or starved vegetative cells (McLean and Pessoney 1971; Pichrtová et al. 2016). These cells differ from younger cells by the accumulation of starch and lipid bodies and reduced chloroplast size. Therefore, we compared the stress resistance of young vegetative cells and old cells. Our old cultures of *S. hirsutum* and *M. thomasiana* (5 months after re-inoculation) resembled “pre-akinetes” of *Zygnema*, which showed similar ultrastructural changes, namely, the accumulation of starch grains and larger lipid bodies containing triacylglycerol. The initial values of Φ_PSII_ differed between old and young cultures for both *S. hirsutum* and *M. thomasiana* strains and were always lower in the old cultures, similar to the *Zygnema* strains (Pichrtová et al. 2014).

In *Micrasterias*, cells that excessively accumulate starch and lipids have been described as “akinetes” or even “winterforms” and have been observed in peatbogs during winter (Meindl et al. 1989; Steiner et al. 2021). These cytologically adapted cells were also observed after artificial cold acclimation in the laboratory. However, they were not able to survive encasement in ice (Steiner et al. 2021), similar to our old, vegetative cells of *M. thomasiana* in the stationary phase, which were no more resistant to desiccation stress than actively growing young cells.

The phenomenon of programmed cell death (PSD) has been described in experimental cultures of *M. denticulata* treated with low concentrations of hydrogen peroxide. Under stress conditions, the death of part of the genetically uniform community can contribute to the long-term survival of the population if the surviving cells can use the resources (nutrients and mucilage) released by the dead cells to protect themselves (Darehshouri et al. 2008). However, the desiccation conditions in our experiments were too severe (all cells died; experiments I and III) or too mild (almost all cells survived, experiment II) to consider PSD. To verify PSD, other hallmarks commonly associated with this process, such as significant increase in caspase-3-like activity, should have been monitored.

We were also able to show that the investigated desmids were unable to acclimate under very mild drought stress. In experiment II, the desiccation process was slowed down by additional moistening of the filters. The samples were re-hydrated after 13 h when the filters appeared dry, but before photosynthesis ceased, both the young and old cultures of *M. thomasiana* and *S. hirsutum* were able to recover their photosynthetic activity after treatment. However, as soon as we prolonged the desiccation time (experiment III, only for old cultures) until Φ_PSII_ dropped (22 h), both species did not recover and died. In contrast, desiccation resistance *Zygnema* was enhanced by cultivation under mild desiccation stress conditions induced either by controlled desiccation at high relative humidity or by pre-cultivation on agar plates. The pre-akinetes could survive severe desiccation (10% RH, desiccation phase lasted 2–10 min) only if they were hardened (Pichrtová et al. 2014). In desmids, on the other hand, prolonged cultivation on wet filters caused stress to the cells and led to a continuous decrease in Φ_PSII_ (experiment IV).

The desiccation rate is generally an important factor affecting survival and recovery after rehydration. For example, the soil filamentous green alga *Klebsormidium dissectum* showed a much faster recovery of photosynthesis when desiccated at 100% RH than at 55% or 5% RH (Karsten and Holzinger 2012). Young cultures of *Zygnema circumcarinatum* exposed to CaCl_2_ (64.5% RH, desiccation phase determined by pronounced drop of Φ_PSII_ lasted 87 ± 28 min) thrived better than cultures desiccated above silica gel (19.5% RH, desiccation phase 79 ± 22 min; Rieseberg et al. 2023). Similarly, *Zygnema* spp. pre-akinetes tolerated conditions of moderate desiccation in most cases (86% RH and slower, desiccation phase lasted tens of minutes to about 5 h). However, the pre-akinetes survived severe desiccation (10% RH, desiccation phase lasted 2–10 min) only if they were pre-cultivated under conditions of mild desiccation stress, “hardening” (Pichrtová et al. 2014). On the other hand, in our experiments, neither young nor old cultures of *S. hirsutum* and *M. thomasiana* survived severe desiccation (75% RH over 5 h, experiment I). However, a slow and mild desiccation treatment of old cultures in the stationary phase (100–75% RH over 22 h, experiment III) also led to cell death. It appears that survival of the desmids studied is determined by a threshold of effective quantum yield rather than desiccation rate. As soon as their Φ_PSII_ drop below a critical value of 0.1, they could no longer recover. However, more experiments are needed to elucidate the effect of desiccation rate on survival of desmids.

During desiccation, light stress could further affect survival and recovery of our strains. There is a significant risk of photodamage during desiccation, as chlorophyll molecules can still become excited when exposed to light, but the energy produced cannot be transferred through photochemical processes. This leads to the production of reactive oxygen species (Gray et al. 2007). During our experiments, continuous illumination was required to allow frequent measurements of the effective quantum yield during the desiccation process. This parameter was measured in a light-adapted state and used as a proxy for photosynthetic activity (Roháček and Barták 1999).

Since *S. hirsutum* was isolated from an ombrogenous pool with a fluctuating water regime, while *M. thomasiana* originated from a minerotrophic pool with a stable water regime, we expected *S. hirsutum* to be more resistant to desiccation stress. Surprisingly, young cultures of both species showed very similar photosynthetic performance, and old cultures also responded similarly to desiccation, both under severe treatment (experiment I) and mild drought stress (experiment II). However, experiment IV indicates that *S. hirsutum* is more resistant. Although most cells died during long-term exposure on a wet filter followed by slow drying, the recultivation test showed that at least some cells of *S. hirsutum* survived this treatment. The drops of treated *S. hirsutum* cultures cut from the filter and placed in an Erlenmeyer flask with fresh medium were able to initiate a new culture, whereas the filter sections with *M. thomasiana* did not. We suspect that this is due to the very firm mucilage that aggregates the cells into macroscopic, densely packed clumps in *S. hirsutum*. The center of the mucilage clump could serve as a refuge where a cell whose Φ_PSII_ does not drop below the critical value (0.1) can survive. On the other hand, the cells of *M. thomasiana* are embedded in a thin layer of mucilage, which forms biofilm on the filter in which the cells do not even overlap and are therefore more susceptible to desiccation. However, further experiments are required to confirm this assumption. The ratio between lipid body and cell content in the 5-month-old cells was also considerably higher in *Staurastrum hirsutum* (28.9%) than in *Micrasterias thomasiana* (15.6%). In addition to the lipid body content and the quantity and quality of mucilage, other adaptive functional traits such as reduced lobulation or a lower surface-to-volume ratio have also been considered to increase resistance of desmid taxa to desiccation (Coesel 1982; Neustupa et al. 2011).

Our results are consistent with the observations of *S. hirsutum* and *M. thomasiana* in different habitats in mountainous peatlands of the Ore Mountains (Czech Republic). *Micrasterias thomasiana* preferred ponds, minerotrophic pools, and streams where the risk of desiccation was close to zero (Neustupa et al. 2023). *Staurastrum hirsutum* was a frequent inhabitant of restored deeper bog pools and recently created drainage channels (Neustupa et al. 2023), and when present in ombrogenous pools, it preferred the deeper pools and clearly avoided those that dried out in summer (Neustupa et al. 2024).

In conclusion, the desmids *Staurastrum hirsutum* and *Micrasterias thomasiana* are not desiccation tolerant, and their desiccation resistance is very limited. It seems that the cells are not able to recover their photosynthetic activity when the effective quantum yield reaches the threshold value and after a few days they die. Based on the recultivation test after long-term exposure on a wet filter and subsequent slow drying, we suppose that *S. hirsutum* is more resistant than *M. thomasiana*, but probably only isolated single cells survived in the middle of macroscopic densely packed clumps of mucilage. Although senescent cells in the stationary phase morphologically resemble the “pre-akinetes” of *Zygnema*, they are obviously not tolerant stages in desmids tested. In nature, at least these two species seem to inhabit localities with a low risk of desiccation or avoid/mitigate desiccation by localized survival strategies, e.g., by mucilage production or, in the case of *Micrasterias*, by directional movement via local mucilage excretion (Oertel et al. 2004).

## Supplementary Information

Below is the link to the electronic supplementary material.

ESM 1 (PNG 669 KB)

High Resolution Image (TIF 3.09 MB)

ESM 2 (PNG 6.29 MB)

High Resolution Image (TIF 37.0 MB)

ESM 3 (PNG 3.06 MB)

High Resolution Image (TIF 14.8 MB)
