# Supplementary figures and images for: Desiccation tolerance in peatland desmids: a comparative study of Micrasterias thomasiana and Staurastrum hirsutum (Zygnematophyceae)
Source: Protoplasma. 2025 Apr 3;262(5):1215–28. doi: 10.1007/s00709-025-02061-1 (PMC12394308; doi:10.1007/s00709-025-02061-1)

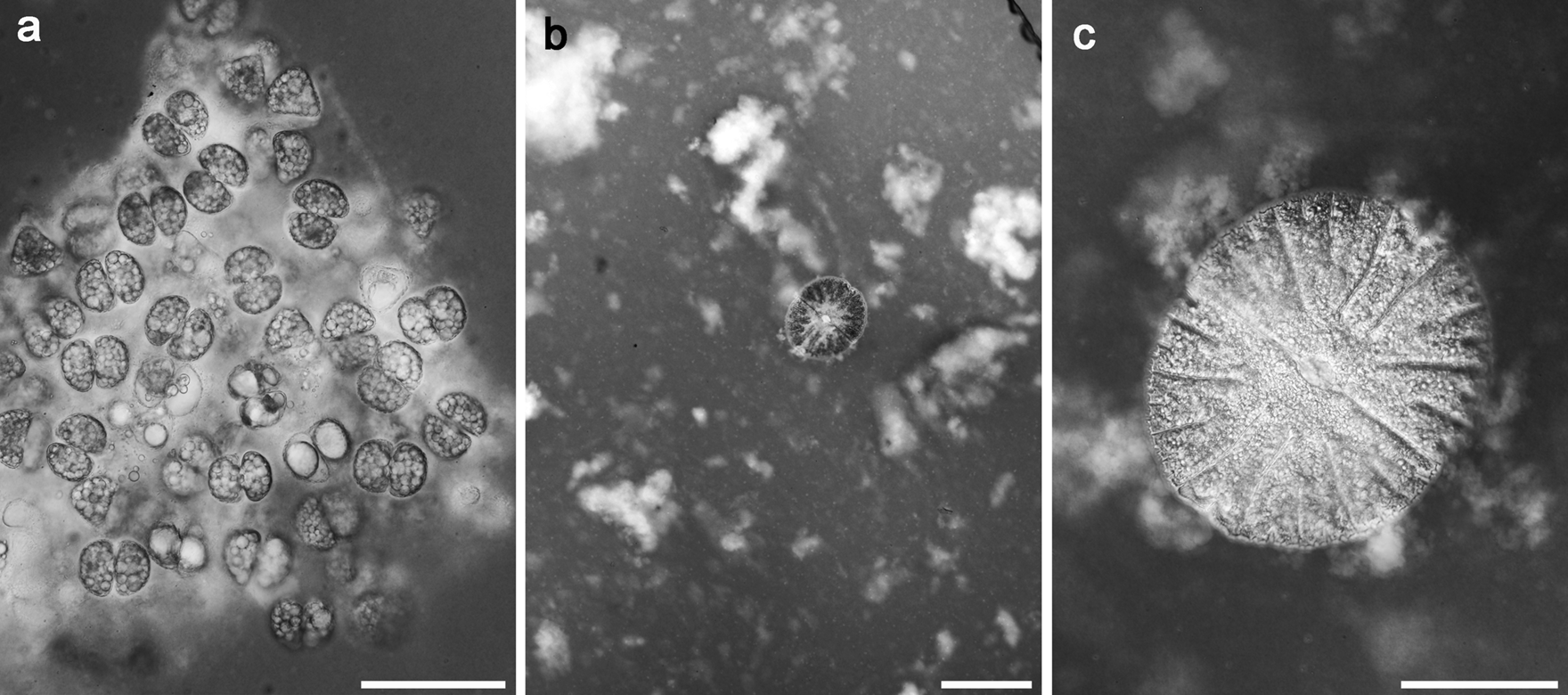

Supplement: Supplementary file 1 — (PNG 669 KB) [file 709_2025_2061_Fig7_ESM.png]

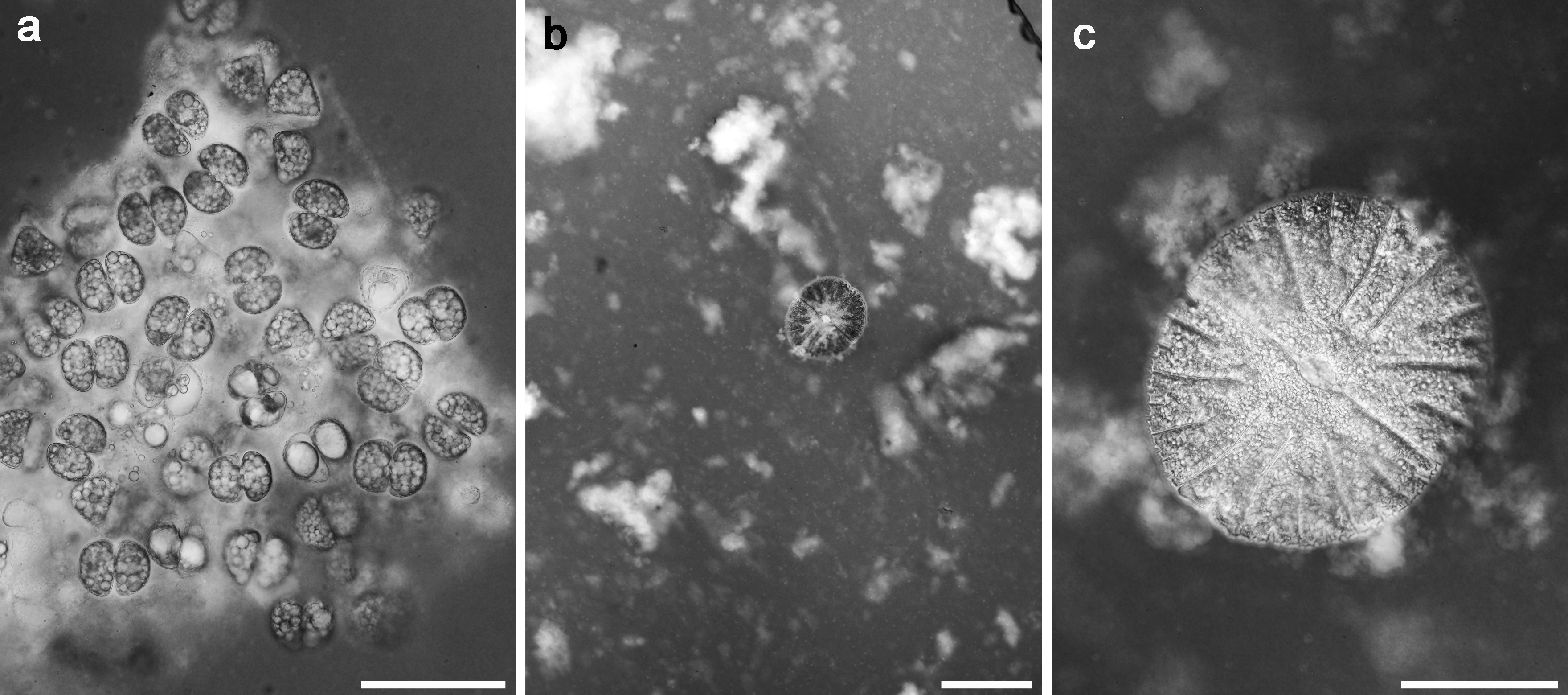

Supplement: Supplementary file 2 — High Resolution Image (TIF 3.09 MB) [file 709_2025_2061_MOESM1_ESM.tif]

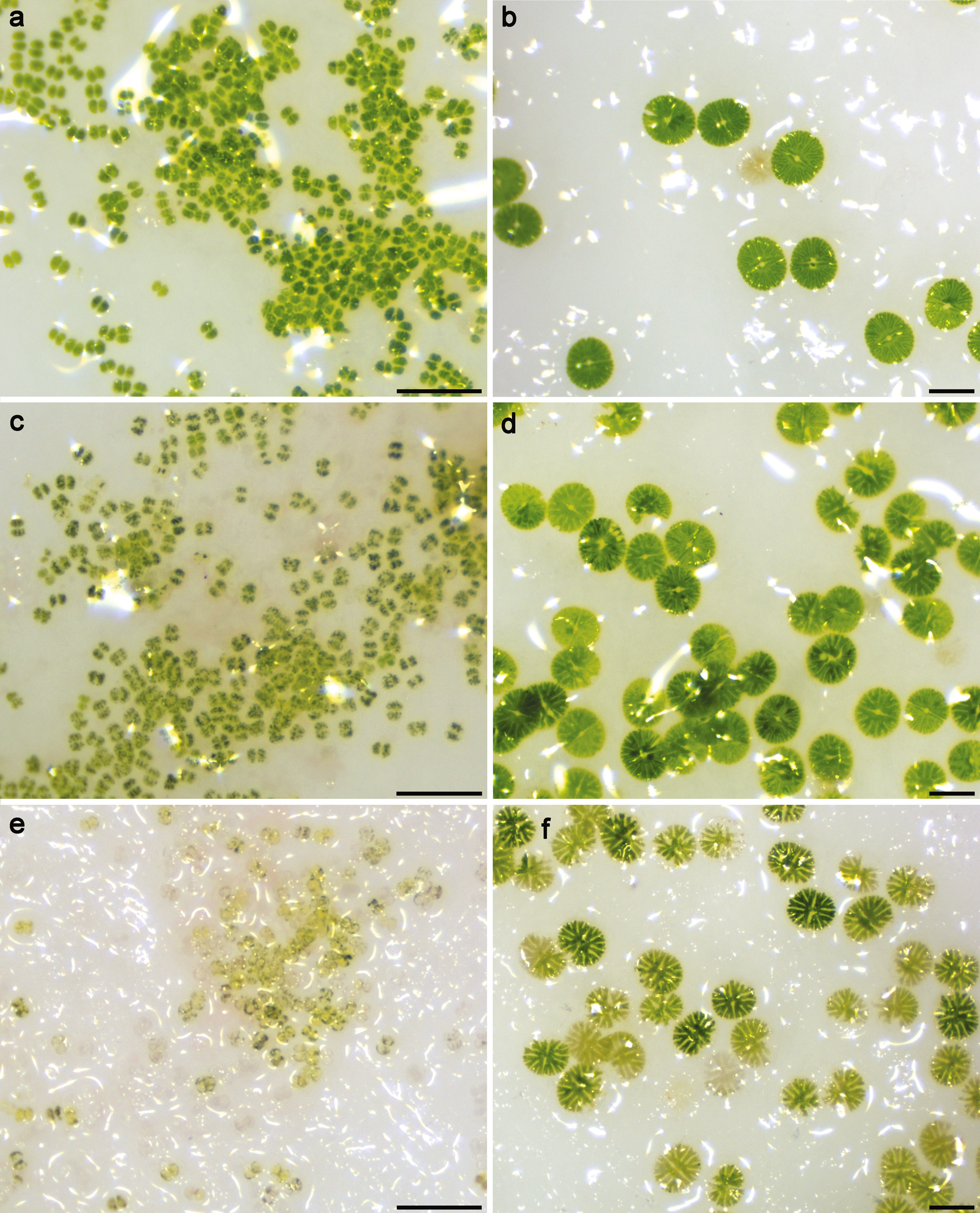

Supplement: Supplementary file 3 — (PNG 6.29 MB) [file 709_2025_2061_Fig8_ESM.png]

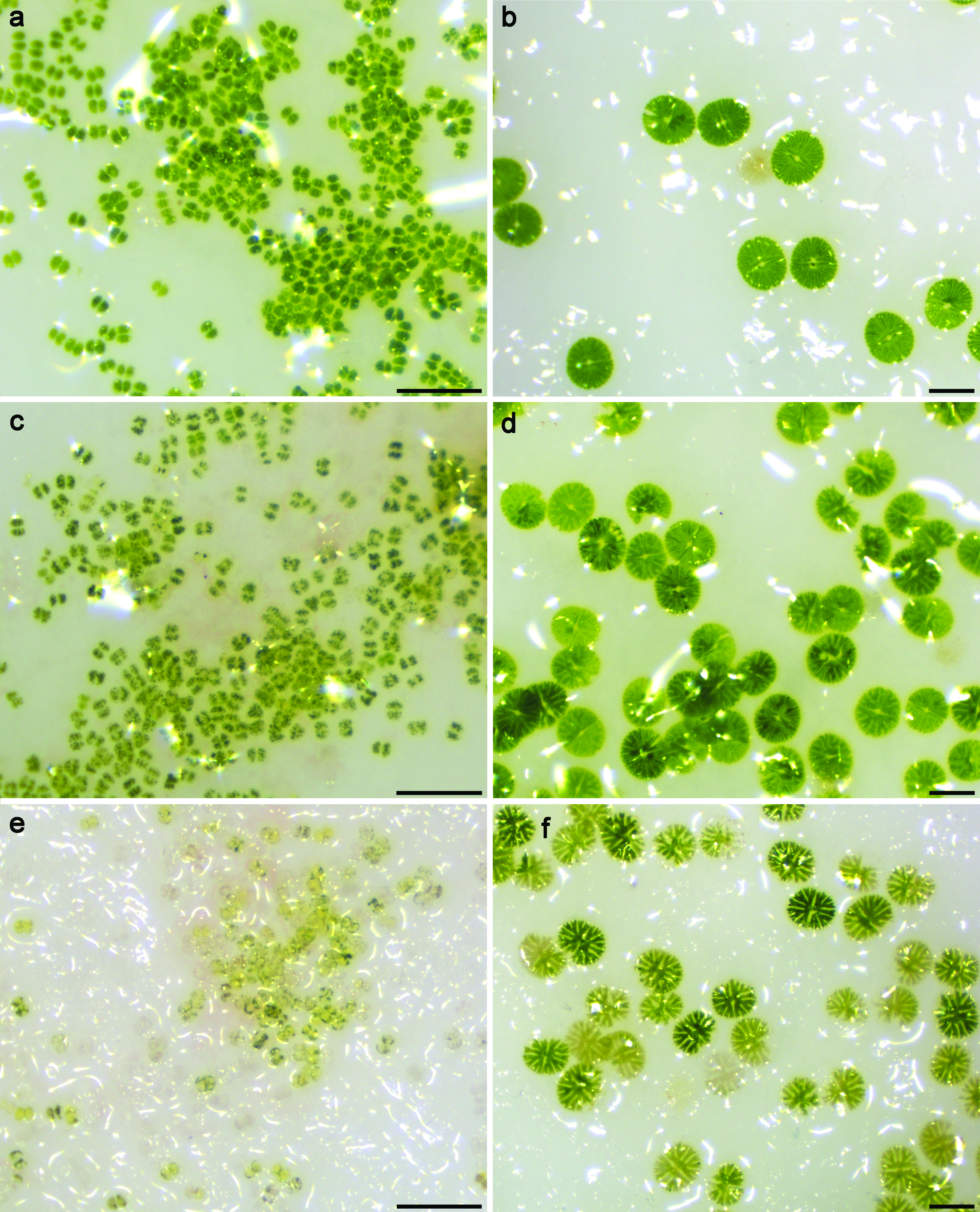

Supplement: Supplementary file 4 — High Resolution Image (TIF 37.0 MB) [file 709_2025_2061_MOESM2_ESM.tif]

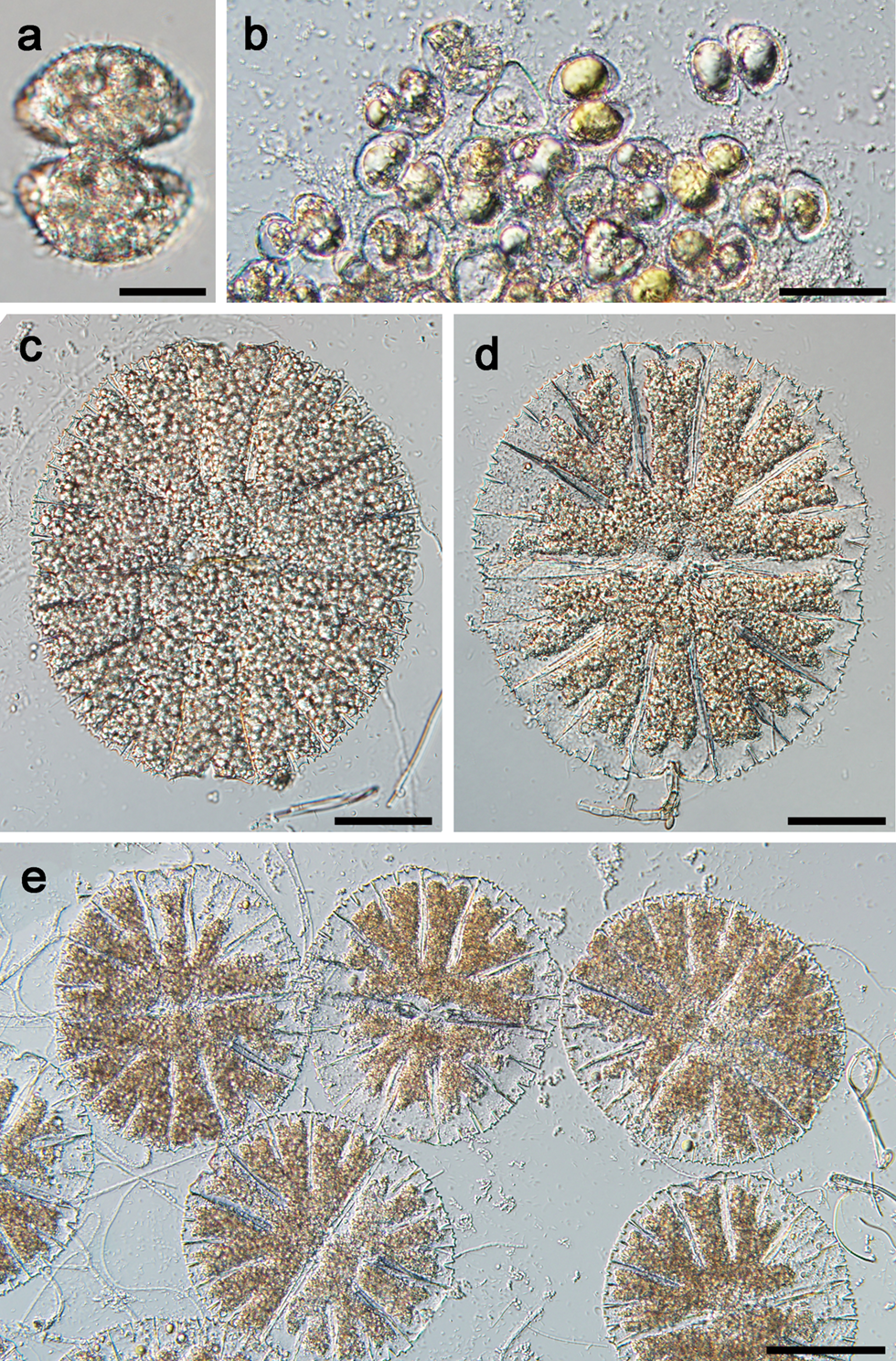

Supplement: Supplementary file 5 — (PNG 3.06 MB) [file 709_2025_2061_Fig9_ESM.png]

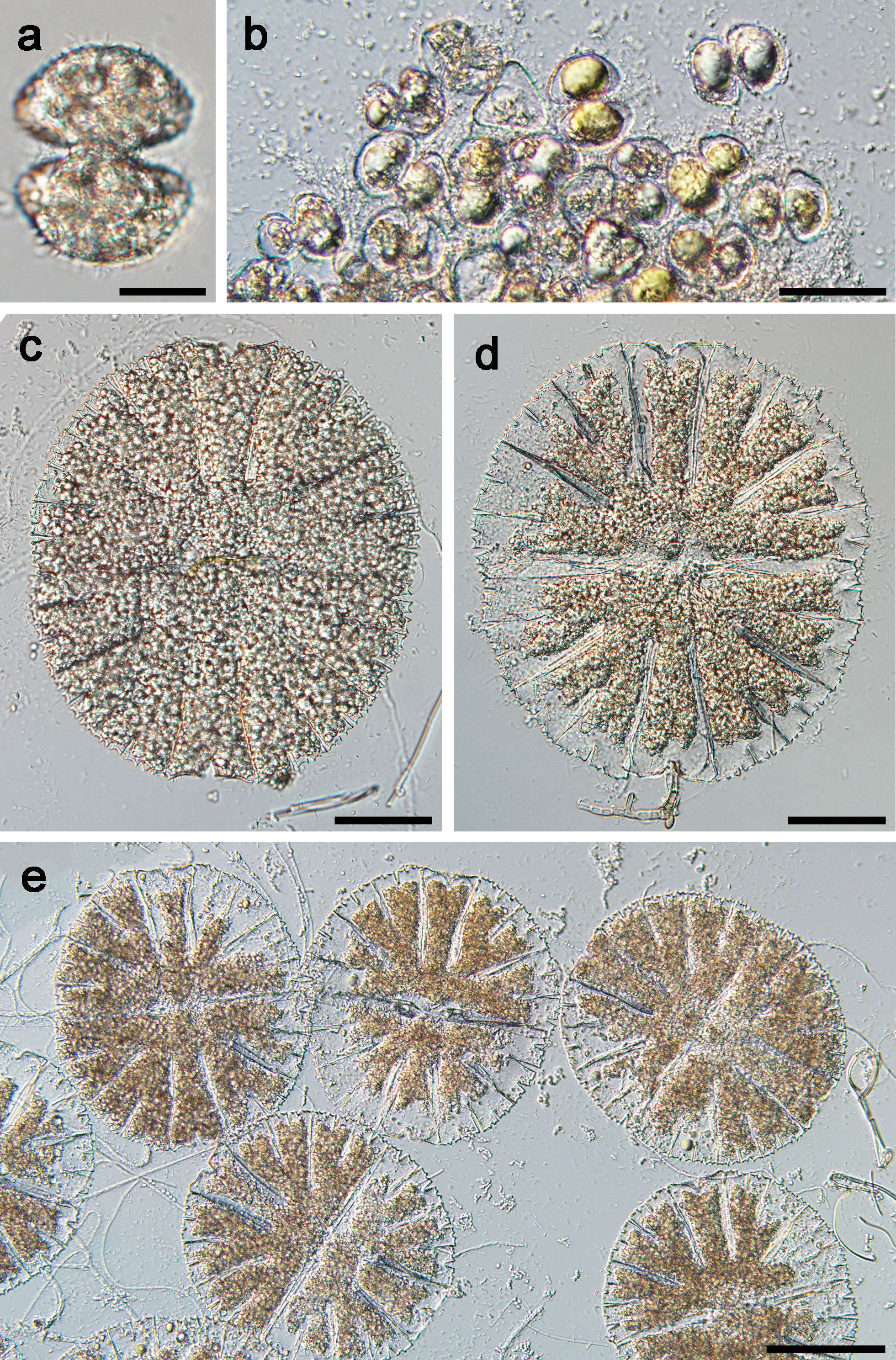

Supplement: Supplementary file 6 — High Resolution Image (TIF 14.8 MB) [file 709_2025_2061_MOESM3_ESM.tif]
